# Analysis of Arterial Stiffness and Sexual Function after Adding on PCSK9 Inhibitor Treatment in Male Patients with Familial Hypercholesterolemia: A Single Lipid Center Real-World Experience

**DOI:** 10.3390/jcm9113597

**Published:** 2020-11-08

**Authors:** Roberto Scicali, Giorgio Ivan Russo, Marina Di Mauro, Flavia Manuele, Grazia Di Marco, Antonino Di Pino, Viviana Ferrara, Agata Maria Rabuazzo, Salvatore Piro, Giuseppe Morgia, Francesco Purrello

**Affiliations:** 1 Department of Clinical and Experimental Medicine, University of Catania, 95100 Catania, Italy; manueleflavia1@gmail.com (F.M.); graziadimarco7@gmail.com (G.D.M.); nino_dipino@hotmail.com (A.D.P.); vivi.fer@hotmail.it (V.F.); rabuazzo@unict.it (A.M.R.); spiro@unict.it (S.P.); francesco.purrello@unict.it (F.P.); 2 Internal Medicine, Garibaldi Hospital, 95122 Catania, Italy; 3 Urology Section, Department of Surgery, University of Catania, 95100 Catania, Italy; giorgioivan1987@gmail.com (G.I.R.); marinadimauro@live.it (M.D.M.); Giuseppe.morgia@unict.it (G.M.)

**Keywords:** familial hypercholesterolemia, PCSK9 inhibitors, arterial stiffness, sexual function, cardiovascular risk

## Abstract

Familial hypercholesterolemia (FH) subjects have high low-density lipoprotein cholesterol (LDL-C) and may be at high risk of erectile dysfunction and atherosclerotic cardiovascular diseases. We evaluated the effect of PCSK9-i on sexual function evaluated by the Male Sexual Health Questionnaire (MSHQ) and the International Index of Erectile Function (IIEF-5) questionnaire and on pulse wave velocity (PWV) in FH male subjects. In this prospective observational study, we evaluated 30 FH male patients on high-intensity statins plus ezetimibe and with an LDL-C off-target. All patients added PCSK9-i treatment and obtained clinical assessment at baseline and after six months of PCSK9-i. As expected, LDL-C significantly decreased after adding-on PCSK9-i (−48.73%, *p* < 0.001). MSHQ and PWV significantly improved after adding-on PCSK9-i (for MSHQ 93.63 ± 6.28 vs. 105.41 ± 5.86, *p* < 0.05; for PWV 9.86 ± 1.51 vs. 7.7 ± 1.42, *p* < 0.05); no significant change of IIEF-5 was found. Finally, a simple regression showed that ∆ MSHQ was significantly associated with ∆ LDL-C and ∆ PWV (*p* value for both <0.05). In conclusion, PCSK9-i therapy significantly improves lipid profile, PWV, and sexual function in FH male patients; our results support the favorable function of PCSK9-i on these parameters.

## 1. Introduction

Atherosclerosis is a continuous and harmful process caused by several etiological factors that favor a persistent vascular injury in the arterial wall [1]. Several studies previously showed that the *primum movens* of atherosclerosis was the endothelial dysfunction that played a crucial role in the dysregulation of the physiological integrity of the arterial wall [2,3]. Moreover, it is of note that the endothelial dysfunction is one of the principal organic causes of erectile dysfunction (ED) defined as the persistent inability to obtain or maintain a satisfactory penile erection during sexual activity [4]. Previous studies showed that the prevalence of ED is increased in male patients with coronary artery disease and ED may be considered an early manifestation of atherosclerotic cardiovascular disease (ASCVD) [5,6]; taking into these considerations, ED and ASCVD may be defined as the two sides of the coin such as systemic vascular burden. In particular, ED is characterized by an impaired endothelium nitric oxide dependent relaxation of cavernous artery, while early atherosclerotic injury is caused by an increased low-density lipoprotein cholesterol (LDL-C) uptake by foam cells in the arterial wall [7,8]. It was previously shown that an incremented LDL-C plasma level promoted the increase of pulse wave velocity (PWV) which was considered an early atherosclerotic injury biomarker and was associated with ASCVD [9,10]; furthermore, LDL-C reduction by statins was associated with PWV improvement [11]. Of note, hypercholesterolemia is frequent in subjects with ED [12]; in this context, subjects with high LDL-C such as familial hypercholesterolemia (FH) may be at high risk of ED and ASCVD.

FH is a genetic disease characterized by high LDL-C levels from an early age [13]. It is the most common monogenic condition and significantly associated with premature ASCVD [14]; thus, early diagnosis and lipid lowering therapies (LLT) are needed for ameliorating cardiovascular prevention in FH patients [15]. However, despite statin therapy, only few FH subjects obtain the adequate lipid targets, while cardiovascular events are present in the majority of FH subjects [16].

In a period of novel treatments, it was an increasing attention regarding the inhibitors of proprotein convertase subtilisin/kexin type 9 (PCSK9-i) [17]. PCSK9-i act to block and inhibit circulating PCSK9 and thus limit LDL receptor cleavage while increasing its presence on the hepatocyte surface [18]. The clinical efficacy of PCSK9-i was shown in previous studies [19,20]; in particular, the reduction of LDL-C and ASCVD by PCSK9-i was ≃50–60% and 15%, respectively. Due to its lipid-lowering efficacy and cardiovascular benefit, PCSK9-i medication is an important additional cure in FH subjects [21].

While previous studies showed that statins could improve sexual function in hypercholesterolemic subjects [22,23], no data exist regarding the effect of PCSK9-i on sexual function in these subjects.

In this study, we aim to evaluate the effect of PCSK9-i on sexual function evaluated by the Male Sexual Health Questionnaire (MSHQ) and the International Index of Erectile Function (IIEF-5) questionnaire and on early atherosclerosis injury evaluated by pulse wave velocity (PWV) in a cohort of FH male subjects.

## 2. Methods

### 2.1. Study Design and Population

This was an open label prospective observational study involving male patients with a previously obtained genetic diagnosis of FH [24]. All participants were enrolled from the Lipid Centre University Hospital of Catania, Italy from September 2018 to May 2020. All participants were aged between 18 and 70 years and had an LDL-C beyond recommended targets despite high-intensity statins (atorvastatin 40–80 mg, rosuvastatin 20–40 mg) plus ezetimibe for at least six months at the time of enrollment. After 12-h fasting, all participants had an assessment of hematological and clinical parameters. For all patients, we obtained biochemical analyses and sexual function and PWV evaluations at baseline (T0) and after six months (T1) of PCSK9-i treatment. Body weight and height were performed, and body mass index (BMI) was calculated as previously performed [25]. Arterial hypertension was defined as previously performed [25]. Statin therapy and its duration was defined as previously performed [26]. Non-statin lipid lowering therapy was defined as previously performed [18]. Type 2 diabetes (T2D) was defined as previously performed [27]. Smoking was defined as previously performed [25]. ASCVD was defined as documented previous myocardial infarction, acute coronary syndrome, coronary revascularization (percutaneous coronary intervention or coronary artery bypass graft surgery) or other arterial revascularization procedures, stroke or transient ischemic attack, or atherosclerotic artery disease [28]. LDL-C target was defined as an LDL-C <70 mg/dl for FH patients without ASCVD or an LDL-C <55 mg/dl for FH patients with ASCVD [29]. Exclusion criteria was the intake of non-statin lipid lowering therapy.

### 2.2. Biochemical Analysis

Fasting plasma glucose (FPG) as well as serum total cholesterol (TC), TG, high-density lipoprotein cholesterol (HDL-C), high sensitivity C-reactive protein (hs-CRP), aspartate transaminase (AST), alanine transaminase (ALT), and creatine phosophokinase (CPK) were measured as previously performed [30]. Apolipoprotein B (ApoB) and apolipoprotein A1 (ApoA1) were evaluated as previously performed [31]. Levels of lipoprotein (a) (Lp(a)) were measured as previously performed [26]. LDL-C was obtained by the Friedewald formula. Non-HDL cholesterol (non-HDL-C) was derived from baseline values. HbA1c was measured as previously performed [32].

### 2.3. Sexual Function Evaluation

To evaluate the erectile and ejaculation functions of the interviewees, we used the following questionnaires: IIEF-5 and MSHQ [33,34]. The IIEF-5 questionnaire was used to evaluate erectile function during the couple relationship. The severity of erectile dysfunction was classified as follows: 25–22 no erectile dysfunction; 21–17 mild erectile dysfunction; 16–12 mild-moderate erectile dysfunction; 11–8 moderate erectile dysfunction; 7–5: severe erectile dysfunction. MSHQ is composed by 25 items with response modalities closed to multiple alternative choice, between five or six proposed options, usually ordered or semi-continuous. It is divided into 5 domains (erectile function (items 1–4); ejaculation and relative pleasure (items 5–12); relationship with the partner (items 13–18); sexual activity in the last month (items 19–21); desire to have sex with fixed partner (item 22–25); and each item scores from 5 (best situation) to 0 (worst situation).

### 2.4. Pulse Wave Velocity Evaluation

The SphygmoCor CVMS (AtCor Medical, Sydney, Australia) system was used for PWV evaluation. This system uses a tonometer and 2 different pressure waves obtained at the common carotid artery (proximal recording site) and at the femoral artery (distal recording site). The distance between the recording sites and the suprasternal notch was measured using a tape measure. An electrocardiogram was used to determine the start of the pulse wave. The PWV was determined as the difference in interval time of the pulse wave between the 2 different recording sites and the heart, divided by the travel distance of the pulse waveform. The PWV was calculated on the mean of 10 consecutive pressure waveforms to cover a complete respiratory cycle [35].

### 2.5. Statistical Analysis

The distributional characteristics of each variable, including normality, were assessed by the Kolmogorov–Smirnov test. Data are reported as mean ± standard deviation (SD) for continuous parametric parameters and median (interquartile range-IQR) for continuous non-parametric variables and as frequency (percentage) for categorical variables. When necessary, continuous non-parametric variables (TG, Lpa, CPK, duration of statin therapy, and hs-CRP) were logarithmically transformed to reduce skewness. The χ2 test was used for categorical variables. The changes of TC, HDL-C, TG, LDL-C, Non-HDL-C, ApoB, ApoAI, ApoB/ApoAI, Lpa, FPG, HbA1c, BMI, systolic BP, diastolic BP, hs-CRP, CPK, GOT, GPT, IIEF-5, MSHQ, and PWV from baseline (T0) to along treatment duration (T1 vs. T0) were evaluated as delta (∆) and calculated according to the following formula: ((T1 − T0)/T0) × 100). To test time-dependent differences (T1 vs. T0) in clinical and biochemical characteristics in the study population, we used Student’s t test. Finally, a simple regression analysis was performed to relate ∆ MSHQ to ∆ LDL-C and ∆ PWV. All statistical analyses were performed using IBM SPSS Statistics for Windows version 23. For all tests, *p* < 0.05 was considered significant.

The study was approved by the local ethics committee (prot. Number 46/19) in accordance with the ethical standards of the institutional and national research committees and with the 1964 Declaration of Helsinki and its later amendments or comparable ethical standards.

Informed consent was obtained from each subject enrolled in the study.

### 2.6. Compliance with Ethical Standards

- *Ethical approval.* This study was approved by the local ethics committee in accordance with the ethical standards of the institutional and national research committees and with the 1964 Declaration of Helsinki and its later amendments or comparable ethical standards. This article does not contain any studies with animals performed by any of the authors.
- *Informed consent.* Informed consent was obtained from each participant enrolled in the study.

## 3. Results

**Figure 1:**
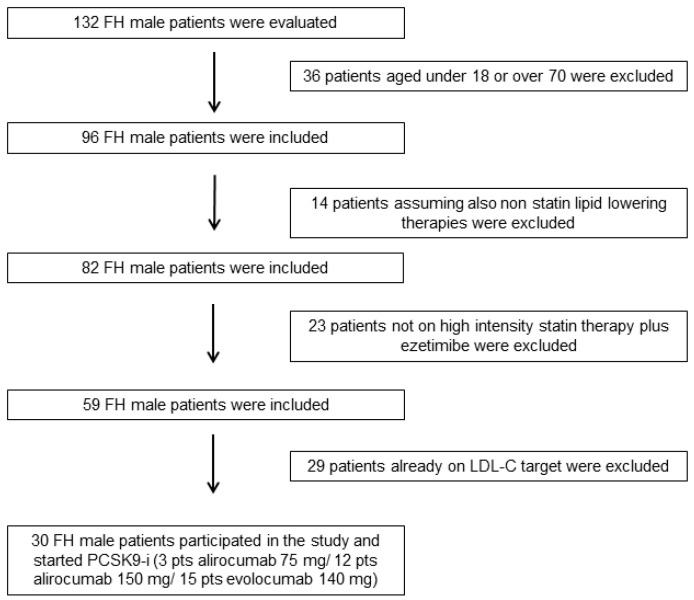
Enrollment flowchart of the study population. FH = familial hypercholesterolemia, PCSK9-i = proprotein convertase subtilisin/kexin type 9 inhibitors.

In total, 132 FH male patients were evaluated; of these, 30 patients satisfied the inclusion criteria and participated to this prospective observational study. According to the lipid lowering recommendation of 2019 European Society of Cardiology/European Atherosclerosis Society guidelines for the management of dyslipidemias and the Italian reimbursement rules of PCSK9-i, all FH patients added PCSK9-i; in particular, 3 patients started alirocumab 75 mg, 12 patients started alirocumab 150 mg, and 15 patients started evolocumab 140 mg (Figure 1).

Table 1 shows the characteristics of the study population; of note, 43.3% of FH patients had a prior ASCVD. All patients were heterozygous FH and the majority of patients had a genetic variant on LDLR; furthermore, more patients were on rosuvastatin 20 mg than atorvastatin 40 mg and 40.0% of patients were on antihypertensive medication.

After 6 months of add-on PCSK9-i therapy, only 43.3% of FH male patients achieved LDL-C targets. As expected, TC, LDL-C, Non-HDL-C, and ApoB plasma levels significantly decreased after adding-on PCSK9-i medication (−34.4%, −48.73%, −43.1%, and −44.16%, respectively), whereas TG, HDL-C, ApoAI, and Lp(a) remained unchanged. Furthermore, the glucose profile was unchanged after adding-on PCSK9-i therapy; moreover, at baseline, 3 FH patients were diabetics and new cases of T2D was not detected after adding-on PCSK9-i therapy. No change of BMI as well as systolic and diastolic BP and hs-CRP were found after adding-on PCSK9-i therapy; furthermore, liver and muscle enzymes were similar before and after PCSK9-i treatment. Concerning sexual function, MSHQ significantly improved after adding-on PCSK9-i therapy (93.63 ± 6.28 vs. 105.41 ± 5.86, *p* < 0.05); finally, IIEF-5 improved after PCSK9-i treatment without reaching statistical significance (Table 2).

**Figure 2:**
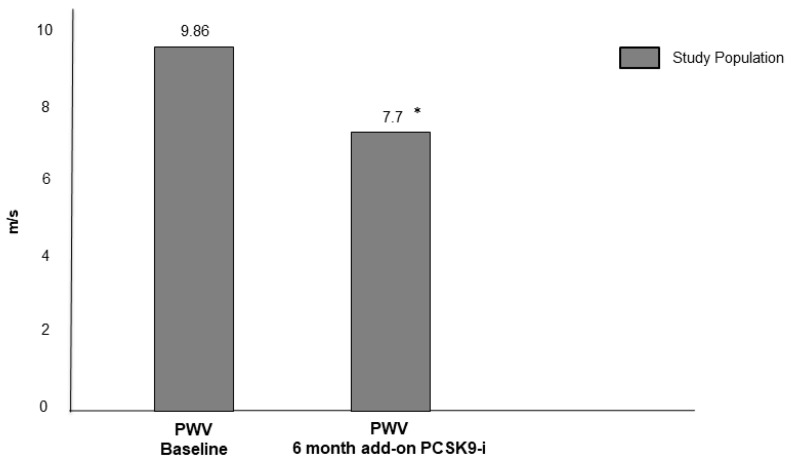
PWV values of the study population after six months of add-on PCSK9-i. PWV = pulse wave velocity, PCSK9-i = proprotein convertase subtilisin/kexin type 9 inhibitors. * *p* value < 0.05 vs. baseline.

As showed in Figure 2, PWV significantly improved after six months of add-on PCSK9-i therapy in FH male patients (9.86 ± 1.51 vs. 7.7 ± 1.42 (∆ −21.9%), *p* < 0.05).

Finally, a simple regression showed that ∆ MSHQ was significantly associated with ∆ LDL-C and ∆ PWV (r = 0.21, *p* value for both <0.05) (Table 3).

## 4. Discussion

In the last years, scientific knowledge has focused on better evaluating the relationship between LDL-C, ED, and ASCVD, and thus the role of lipid control in high cardiovascular risk patients such as FH obtained growing consideration. ED as well as ASCVD may be considered as two different conditions of the same vascular burden and are related to LDL-C levels [36,37]; in this context, novel lipid lowering therapies such as PCSK9-i may be useful to reduce the double vascular burden of FH male patients.

In our study, we evaluated the impact of PCSK9-i on lipid profile, sexual function, and PWV in FH male patients; to the best of our knowledge, this is the first study evaluating the role of PCSK9-i on both sexual function and arterial stiffness. We found that LDL-C and PWV were significantly reduced after six months of adding-on PCSK9-i and MSHQ significantly improved after this treatment; moreover, a simple regression showed that ∆ MSHQ was significantly associated with ∆ LDL-C and ∆ PWV.

It is of note that statin significantly improved lipid profile, sexual function, and PWV in the general population [11,38,39]; for these reasons, statin is the first LDL-C lowering choice in the general population, in particular, in FH subjects. However, despite statins, FH patients continued to experience premature ASCVD [40]. Therefore, because early and better reduction of LDL-C could significantly reduce cardiovascular risk, novel lipid-lowering therapies such as PCSK9-i may be useful to reduce LDL-C levels and thus the vascular burden of FH patients. Concerning LDL-C reduction by PCSK9-i, we found a significant LDL-C reduction of 49%; this is in line with previous findings by Hollstein et al. who found a similar LDL-C reduction in a real world setting of patients at high cardiovascular risk [41]. Concerning PWV reduction by PCSK9-i, our findings are in line with previous studies that showed a similar effect [42,43]. In fact, Mandraffino et al. recently showed that PCSK9-i significantly reduced PWV compared to ezetimibe in a cohort of FH subjects with or without ASCVD; furthermore, Cicero et al. found a significant reduction of PWV already after three months of PCSK9-i medication. PWV is a modern cardiovascular biomarker independently associated to ASCVD and mortality and largely performed in clinical practice. In particular, it was shown that the traditional cardiovascular risk factors are correlated to a deleterious increment of PWV which has a strong predictive value for cardiovascular morbidities in the general population [44]. Of note, Kumagai et al. previously showed that PWV was significantly associated with sexual function in a cohort of dysmetabolic male patients [45]; in line with this finding, our study showed that ∆ PWV was significantly associated to ∆ MSHQ. MSHQ is a validated questionnaire able to detect an unsatisfactory sexual function in general population; of note, Vlachopoulos et al. showed that ED evaluated by MSHQ and IIEF-F was significantly associated with an increased risk of ASCVD and mortality in the general population [46]. In this context, an improvement of sexual function may be useful to reduce cardiovascular risk in male patients; in line with this consideration, in our study, the improvement of sexual function by PCSK9-i was associated with a reduction of cardiovascular risk evaluated by PWV.

There are several limitations to our study; first, the present study was an open label prospective observational but not randomized study and the PCSK9-i therapeutic choice depended on physician judgment. Moreover, ED was only evaluated by questionnaires, while ultrasonography evaluation of penis was not performed. Furthermore, other parameters that may explain the interaction of LDL-C, PWV, and ED such as flow mediated dilation, nitrite, and oxidative stress determinations were not performed. Data on specific antihypertensive treatments were not available. Study population size was relatively small; however, we showed a significant improvement of lipid profile, PWV, and MSHQ after PCSK9-i in FH male patients. Of course, these preliminary findings should be further confirmed in a wider study population through appropriate diagnostic tools and statistical analyses. In conclusion, PCSK9-i therapy significantly improved lipid profile, PWV, and sexual function in FH male patients; moreover, ∆ MSHQ was significantly associated with ∆ LDL-C and ∆ PWV. Our results support the beneficial role of these novel, non-statin, lipid-lowering therapies on sexual function and PWV; however, a randomized controlled trial is needed to assess the impact of PCSK9-i on these parameters in FH male patients.

## Figures and Tables

**Table 1:** Characteristics of the study population.

Demographic Characteristics	
***n***	30
Age, years	55.69 ± 8.89
Men, *n* (%)	30 (100)
Smoking, *n* (%)	11 (36.7)
ASCVD, *n* (%)	13 (43.3)
**FH Genotype**	
LDLR, *n* (%)	29 (96.7)
ApoB, *n* (%)	1 (3.3)
**Mutation class**	
Aminoacid change, *n* (%)	17 (56.7)
Null allele, *n* (%)	13 (43.3)
**FH Phenotype** Heterozygous FH, *n* (%)	30 (100)
**Treatment**	
Duration of statin therapy, years	11 (2–19)
Ezetimibe, *n* (%)	4 (1–7)
Antihypertensive therapy, *n* (%)	12 (40.0)
**High Intensity Statin Therapy**	
Atorvastatin 40 mg, *n* (%)	12 (40.0)
Rosuvastatin 20 mg, *n* (%)	18 (60.0)

Data are presented as mean ± standard deviation, percentages, or median (interquartile range). FH = familial hypercholesterolemia, ASCVD = atherosclerotic cardiovascular disease, LDLR = low-density lipoprotein receptor, ApoB = apolipoprotein B.

**Table 2:** Glucose, lipid, risk factor, and sexual function profiles of the study population at baseline and after six months of add-on PCSK9-i.

	FH Male Subjects (*n* = 30)	FH Male Subjects (*n* = 30)	∆	*p* Value between the Two Groups
	Baseline	6-Month Add-on PCSK9-i		
**Glucose Profile**				
FPG, mg/dL	95.45 ± 8.56	93.44 ± 7.83	−2.11%	0.73
HbA1c, %	5.75 ± 0.48	5.8 ± 0.43	0.87%	0.71
Type 2 Diabetes, *n* (%)	3	3	-	-
**Lipid Profile**				
TC, mg/dL	212.05 ± 17.97	139.1 ± 17.32	−34.4%	<0.001
HDL, mg/dL	46.82 ± 8.28	48.56 ± 7.74	3.71%	0.46
TG, mg/dL	88.5 (60.5–120)	87 (60.25–110.25)	−1.69%	0.63
LDL-C, mg/dL	145.65 ± 17.04	74.67 ± 16.91	−48.73%	<0.001
LDL-C target, *n* (%)	-	13 (43.3)	-	-
Non-HDL-C, mg/dL	164.23 ± 16.98	93.44 ± 16.94	−43.1%	<0.001
ApoB, mg/dL	108.82 ± 17.24	60.76 ± 17.45	−44.16%	<0.001
ApoAI, m g/dL	130.13 ± 17.18	133.21 ± 17.77	2.37%	0.66
ApoB to ApoAI ratio	0.91 ± 0.19	0.47 ± 0.18	−48.35%	<0.001
Lp(a), nmol/L	21.9 (11.1–44.7)	15.4 (9.7–33.7)	−29.68%	0.15
**Risk Factors**				
Body mass index, kg/m^2^	26.25 ± 2.23	26.1 ± 2.17	−0.57%	0.86
Systolic BP, mmHg	120.23 ± 9.94	117.05 ± 10.01	−2.64%	0.34
Diastolic BP, mmHg	72.5 ± 10.09	71.6 ± 10.3	−1.24%	0.67
hs-CRP, mg/dL	0.16 (0.07–0.34)	0.14 (0.06–0.28)	−14.28%	0.87
**Liver and Muscle Enzymes**				
AST, U/L	28.59 ± 8.03	26.05 ± 8.31	−8.88%	0.33
ALT, U/L	32.29 ± 9.28	30.06 ± 9.16	−6.91%	0.42
CPK, U/L	118 (83.0–156.5)	114 (81–152.25)	−3.39%	0.81
**Sexual Function**				
MSHQ	93.63 ± 6.28	105.41 ± 5.86	12.58%	<0.05
IIEF-5	20.92 ± 2.28	24.45 ± 2.34	16.87%	0.06

Data are presented as mean ± standard deviation, percentages, or median (interquartile range). PCSK9-i = proprotein convertase subtilisin/kexin type 9 inhibitors, FH = familial hypercholesterolemia, FPG = fasting plasma glucose, HbA1c = glycated hemoglobin, TC = total cholesterol, HDL = high-density lipoprotein, TG = triglycerides, LDL = low-density lipoprotein, TG/HDL = triglyceride to high-density lipoprotein ratio, ApoB = apolipoprotein B, ApoAI = apolipoprotein AI, Lp(a) = lipoprotein (a), hs-CRP = high sensitivity C-reactive protein, BP = blood pressure, AST = aspartate transaminase, ALT = alanine transaminase, CPK = creatine phosophokinase, MSHQ = Male Sexual Health Questionnaire, IIEF-5 = International Index of Erectile Function.

**Table 3:** Simple linear regression analyses evaluating ∆ MSHQ as dependent variable.

Dependent Variable	∆ MSHQ	
Independent Variable	Coefficient β	*p* Value
∆ LDL-C, %	1.628 ± 0.175	<0.05
∆ PWV, %	1.251 ± 0.111	<0.05

∆ MSHQ = change of MSHQ from baseline to along PCSK9-i therapy duration, ∆ LDL-C = change of LDL-C from baseline to along PCSK9-i therapy duration, ∆ PWV = change of PWV from baseline to along PCSK9-i therapy duration.
